# Insights into the microbiota of raw milk from seven breeds animals distributing in Xinjiang China

**DOI:** 10.3389/fmicb.2024.1382286

**Published:** 2024-10-23

**Authors:** Baolong Luo, Fujin Dong, Yuyang Liu, Jie Du, Hailong Sun, Yongqing Ni, Yan Zhang

**Affiliations:** ^1^Key Laboratory of Xinjiang Special Probiotics and Dairy Technology of Shihezi Municipal Government, School of Food Science and Technology, Shihezi University, Shihezi, Xinjiang, China; ^2^Xinjiang Production and Construction Corps Industrial Innovation Research Institute of Dairy Products, Xinjiang Tianrun Dairy Co., Ltd., Urumchi, Xinjiang, China; ^3^Key Laboratory of Agricultural Product Processing and Quality Control of Specialty (Co-construction by Ministry and Province), School of Food Science and Technology, Shihezi University, Shihezi, Xinjiang, China; ^4^Key Laboratory for Food Nutrition and Safety Control of Xinjiang Production and Construction Corps, School of Food Science and Technology, Shihezi University, Shihezi, Xinjiang, China

**Keywords:** raw milk, breeds, regions, bacterial, community structures

## Abstract

Owing to its high nutritional content, raw milk contains a rich microbiota. Thus, to study microorganisms present in raw milk available in Xinjiang China, 142 raw milk samples from seven animal breeds (cow, sheep, goat, donkey, horse, camel, and yak) and four regions (Hami, Tarbagatay, Kashgar, and Ili) were analyzed by high-throughput DNA sequencing. These microorganisms were characterized by 10 dominant phyla. Proteobacteria (68.33%) was the major phylum, followed by Firmicutes (18.80%) and Thermi (3.16%). Horse milk contained more Bacteroidetes, sheep milk contained more Gammaproteobacteria, and donkey milk contained more unclassified sequences. Camel and donkey milk contained the highest and lowest bacterial diversity compared with that contained by the remaining milk samples, respectively. Additionally, spoilage microorganisms, including *Chryseobacterium*, *Propionibacterium*, and *Flavobacterium*, and pathogenic bacteria, including *Ochrobactrum anthropi* and *Sphingomonas*, were more prevalent in horse and yak milk, whereas probiotic lactic acid bacteria (LAB), such as *Leuconostoc*, *Lactococcus*, or *Lactobacillus*, were more prevalent in goat, donkey, and camel milk. Furthermore, *Moraxella* was abundantly present in goat, camel, and yak milk, *Acinetobacter* was more abundant in camel milk, and *Pseudomonas* was relatively abundant in sheep and donkey milk. Overall, specific harmful microorganisms and probiotic lactic acid bacteria were found in the raw milk samples obtained from different animals, which provided a basis for preventing and controlling the growth of harmful bacteria, as well as investigating probiotic resources in raw milk.

## 1 Introduction

The vast Xinjiang Uygur Autonomous Region of China boasts an expansive terrain and abundant grassland resources, fostering a rich heritage of free-range animal husbandry across its diverse regions such as Ili, Hami, Tarbagatay, and Kashgar (Li et al., 2012). This practice, underpinned by the region’s unique climatic and environmental endowments, has nurtured a diverse array of livestock including horses, cow, yaks, goats, sheep, camels, and donkeys. Concurrently, the various kinds of animal milk and derivative products—encompassing yogurt, cheese, koumiss, and ghee, among others—are not only rich in nutrients but also distinguished by their diverse flavors, earning the profound affection of both locals and tourists alike (Mo et al., 2019). Furthermore, owing to the constraints of production environments in pasture, the majority of the gathering and processing of animal raw milk is conducted manually by herdsmen, resulting in a significant enrichment of indigenous microorganisms within the milk (Wouters et al., 2002). These microorganisms are closely related to the nutritional value, processing capabilities, storage stability, and ultimately, the health benefits imparted to consumers of raw milk (Panesar, 2011; Quigley et al., 2013). In essence, they form the cornerstone of a robust and interconnected system that underscores the unique qualities of the region’s dairy heritage.

Previous research endeavors, employing culture-dependent and/or culture-independent methods, have consistently highlighted the composition and characteristics of beneficial microorganisms in animal raw milk and dairy products (Dalmasso et al., 2017; Zhang et al., 2019; Kamilari et al., 2020; Zhu et al., 2020; Chi et al., 2021; Rahmeh et al., 2022; Rajawardana et al., 2022; Santamarina-García et al., 2022). These microorganisms predominantly encompass lactic acid bacteria (LAB) such as *Lactobacillus* (Mahmoudi et al., 2016), *Bifidobacterium* (Yasmin et al., 2020), *Lactococcus* (Kondrotiene et al., 2020), *Streptococcus* (Ayyash et al., 2018), *Leuconostoc* (Ariute et al., 2023), Pediococcus (Moussaid et al., 2023), Propionibacterium (Yerlikaya et al., 2020) and Corynebacterium (Hahne et al., 2018) along with yeast (Zhang et al., 2021) and mold (Quigley et al., 2013). Notably, these microorganisms significantly contribute to enhancing the flavor profile, texture, and nutritional composition of raw milk and dairy products (Karahadian et al., 1985; Masoud and Jakobsen, 2005; Moosavi-Nasab et al., 2010; Thierry et al., 2011; Hahne et al., 2018). Nevertheless, studies have revealed the coexistence of spoilage and pathogenic microorganisms within raw milk and derived products pose a significant challenge (Hassan and Frank, 2011). Specifically, the heat-resistant microorganisms (primarily *Bacillus* species) (Yang et al., 2023), psychrotolerant and/or psychrophilic microorganisms, particularly *Pseudomonas*, *Acetobacter*, and *Aeromonas*, resulted in the deterioration of raw milk and processed products during processing and storage stages, causing considerable disruptions to human production processes (Nörnberg et al., 2010; Samaržija et al., 2012; Xin et al., 2017; Yang et al., 2020). Furthermore, the detection of pathogenic microorganisms like *Staphylococcus*, *Campylobacter*, *Yersinia*, *Salmonella*, *Escherichia coli*, *Listeria*, *Brucella*, *Aeromonas*, *Bacillus*, *Clostridium*, *Serratia*, and *Proteus* in animal raw milk or derivatives underscores the potential food safety hazards and associated risks to human health (Gran et al., 2003; Jin et al., 2009; Fotou et al., 2011; Quigley et al., 2013; Verraes et al., 2014; Jamali et al., 2015; Fei et al., 2019).

Currently, amidst the advancements in dairy industrialization, the processing of raw animal milk in pastoral regions has undergone a paradigm shift, transitioning from manual methods to centralized factory operations, where it is transformed into a diverse array of standardized dairy products. Consequently, there arises a paramount need for a comprehensive understanding of the microbial composition and safety assessment of raw milk sourced from various animal species (Wu et al., 2018). Illumina MiSeq high-throughput sequencing technology allows for more comprehensive and accurate detection of species composition compared to traditional culture-dependent methods (Sessou et al., 2019). In this study, a comparative analysis of the microbiota in animal raw milk from 142 fresh samples, collected from seven diverse animal species (cow, sheep, goat, donkey, horse, camel, and yak) in four representative pastoral areas (Hami, Tuscaloosa, Kashgar, and Ili) of Xinjiang, China, was presented using high-throughput sequencing technologies targeting the V3–V4 hypervariable region of the 16S ribosomal RNA gene. To delve deeper into the differences and interrelationships among microorganisms present in raw milk from diverse animals, we employed Linear Discriminant Analysis Effect Size (LEfSe) alongside Indicator Species Analysis. Our research objective is to assess the potential beneficial microbial resources in animal raw milk and the safety of dairy products, so as to provide theoretical basis for subsequent production, processing and animal breeding.

## 2 Materials and methods

### 2.1 Sample collection

In total, 142 raw milk samples collected from cows (N), sheep (MY), goats (SY), donkeys (L), horses (M), camels (T), and yaks (MN) in the regions of Hami (HM), Tarbagatay (TC), Kashgar (KS), and Ili (YL) in Xinjiang, China. The samples were collected in sterilized tubes from local herding families living in the four regions of Xinjiang, China, and transferred to the laboratory using a mobile refrigerator (operating at −18 to −15°C) to be stored at −80°C for further analyses.

### 2.2 DNA extraction and high-throughput sequencing

One milliliter of the milk was centrifuged at 10000 *g* for 10 min to obtain a pellet, which was subjected to DNA extraction. Total DNA was extracted from each milk sample using PowerSoil DNA Isolation Kit (MoBio, Carlsbad, CA, United States) according to the manufacturer’s instructions. The obtained DNA was quantified using PicoGreen (Invitrogen, Carlsbad, CA, United States) and stored at −20°C. Further, a DNA solution adjusted to 10 ng DNA/μL in H_2_O was pretreated with 1 μg BSA/mL (BSA concentration in the sample: 10 mg/mL) at 95°C for 5 min to bind to polymerase chain reaction (PCR)-inhibiting substances. Next, 16S rRNA gene libraries were constructed by performing PCR to amplify the variable regions V3 and V4 using the forward 16Sf (5′-CCTACGGGAGGCAGCAG-3′) and the reverse 16Sr (5′-GGACTACHVGGGTWTCTAAT-3′) primers (Zhang et al., 2019).

After the quantification, qualification, and purification of the PCR products, a sequencing library was developed using NEB Next R UltraTM DNA Library Prep Kit for Illumina (NEB, Ipswich, MA, United States). The library was sequenced using the Illumina HiSeq 2000 system (Illumina, Inc., San Diego, CA, United States), which generated 300-bp paired-end reads.

### 2.3 Sequence analyses

The quality control of the resulting bacterial reads was performed according to Ben Maamar et al. (2020). Briefly, sequences with barcode ambiguities, with read length < 150 bp, and with average quality score < 25 were removed. Uchime was used to remove chimeric sequences (Edgar et al., 2011). Subsequently, the processed sequences were clustered in operational taxonomic units (OTUs) defined at 97% similarity using CROP (Hao et al., 2011). Taxonomic analyses were conducted using MOTHUR by querying the bacterial and archaea reads against those in the GREENGENES (Ben Maamar et al., 2020) reference databases.

### 2.4 Statistical analyses

#### 2.4.1 Indices of *α*-diversity

Simpson, Chao, Ace, and Shannon diversity indices, which are indices of α-diversity, were estimated for total bacteria based on OTU abundance matrices rarefied to the lowest sequence numbers. The α-diversity indices were analyzed by QIIME with the MOTHUR function. The one-way analysis of variance and Pearson’s correlation analysis were performed using R 3.5.1 statistical software (R Development Core Team, 2013). Tukey’s honest significant difference test was used to determine differences among α-diversity indices. The results were considered significantly significant at *p* < 0.05.

#### 2.4.2 Principal coordinate analyses

The overall variability in bacterial community structures was evaluated by performing PCoA using the *procrustes* function as implemented in the *vegan* package in R. The *ggplot2* package with *ggscreeplot*v and *ggbiplot* functions were used to replace the built-in R function *biplot.princomp* with extended functionality for labeling groups.

#### 2.4.3 Correlation coefficient analysis

The correlation coefficients among bacteria were determined using R3.5.1 with *corrgram* and *ggplot2* packages and *lattice*, *survival*, *Formula*, *Hmisc*, and *corrgram* functions. The results were visualized using Cytoscape.

#### 2.4.4 LEfSe

Significant taxonomic differences were analyzed by performing the LEfSe analysis (Segata et al., 2011). Significant taxa were used to indicate differences among the sample (Bokulich et al., 2014). LAD scores were normalized by log10.

#### 2.4.5 Indicator species analysis

Indicator species analysis was performed using the *multipatt* function in the *indicspecies* package in R, allowing 999,99 permutations and combinations between habitats (De Cáceres et al., 2010) to identify OTUs that caused changes in multivariate patterns. Multiple testing corrections of *p*-values were performed in R using the *fdrtool* function in the *fdrtool* package (Strimmer, 2008). All graphs were generated with R using the *vegan* (Oksanen et al., 2013) and *ggplot2* packages (Wickham, 2009). Only OTUs with significant INDicator VALues (INDVAL) (*p* < 0.05) more than 0.3 were considered and visualized by the matrix–bubble graph using *ggplot2* in R3.5.1 with the *grid*, *showtext*, and *Cairo* functions.

INDVAL were analyzed depending on two site-group combinations as follows: (1) Seven-group combination; N (cows), MY (sheep), SY (goats), L (donkeys), M (horses), T (camels), and MN (yaks) and (2) Four-group combination; HM (Hami), TC (Tarbagatay), KS (Kashgar), and YL (Ili).

## 3 Results

### 3.1 Compositions of microbial communities in milk samples

**Figure 1:**
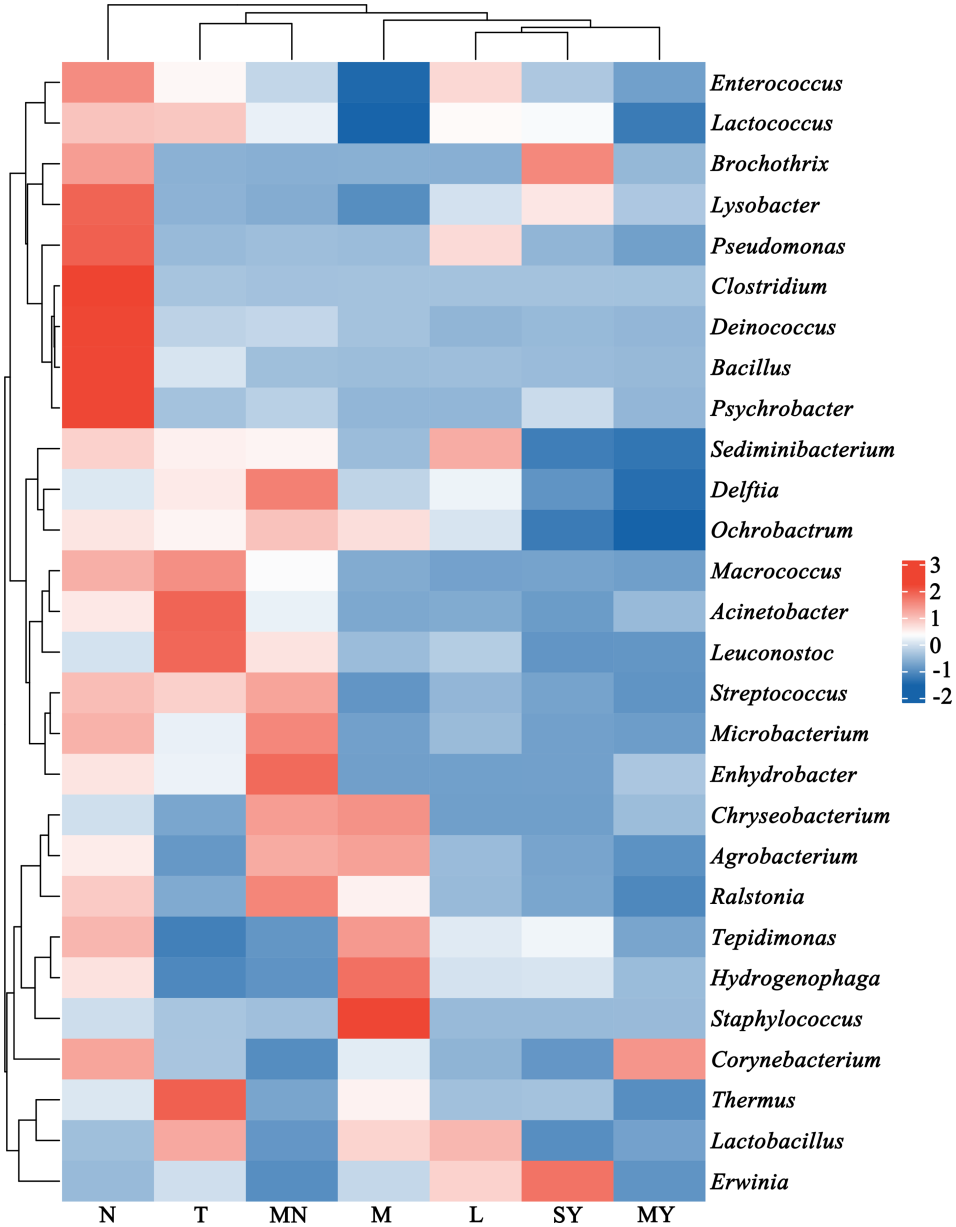
Community structure of the dominant bacteria in 142 raw milk samples of seven different animal breeds at the phylum level (relative abundance >1%). L, donkey milk; M, horse milk; MN, yak milk; MY, sheep milk; N, cow milk; SY, goat milk; T, camel milk.

In total, 128,607 reads were obtained in the present study. The number of effective reads per sample ranged from 30,693 to 44,837. The microbial communities were characterized by six dominant phyla (>1.0% of the total sequences) and 13 less abundant phyla representing 99.72 and 0.28% of the total sequences, respectively, whereas 4.90% of the sequences were unclassified at the phylum level. Proteobacteria was the major phylum with a relative abundance of 68.33%, followed by Firmicutes (18.80%), Thermi (3.16%), Bacteroidetes (2.35%), and Actinobacteria (1.83%). Among Proteobacteria, Gammaproteobacteria was the most abundant class (86.20% of the Proteobacteria sequences), followed by Alphaproteobacteria (7.45%), Betaproteobacteria (6.29%), and Deltaproteobacteria (0.06%). The milk samples were classified into groups N (cows), MY (sheep), SY (goats), L (donkeys), M (horses), T (camels), and MN (yaks) according to their breeds. The relative abundances of phyla in each of these groups are presented in Figure 1. The relative abundance of unclassified sequences at the phylum level was more than that reported in previous studies (Hou et al., 2015; Doyle et al., 2017). Moreover, 74.37% of these unclassified sequences were from the donkey milk samples. The camel milk samples were shown to contain abundant new species. The relative abundance of bacteroidetes in the horse milk samples was significantly higher than that in the remaining milk samples. Gammaproteobacteria was relatively abundant in the sheep milk samples.

**Figure 2:**
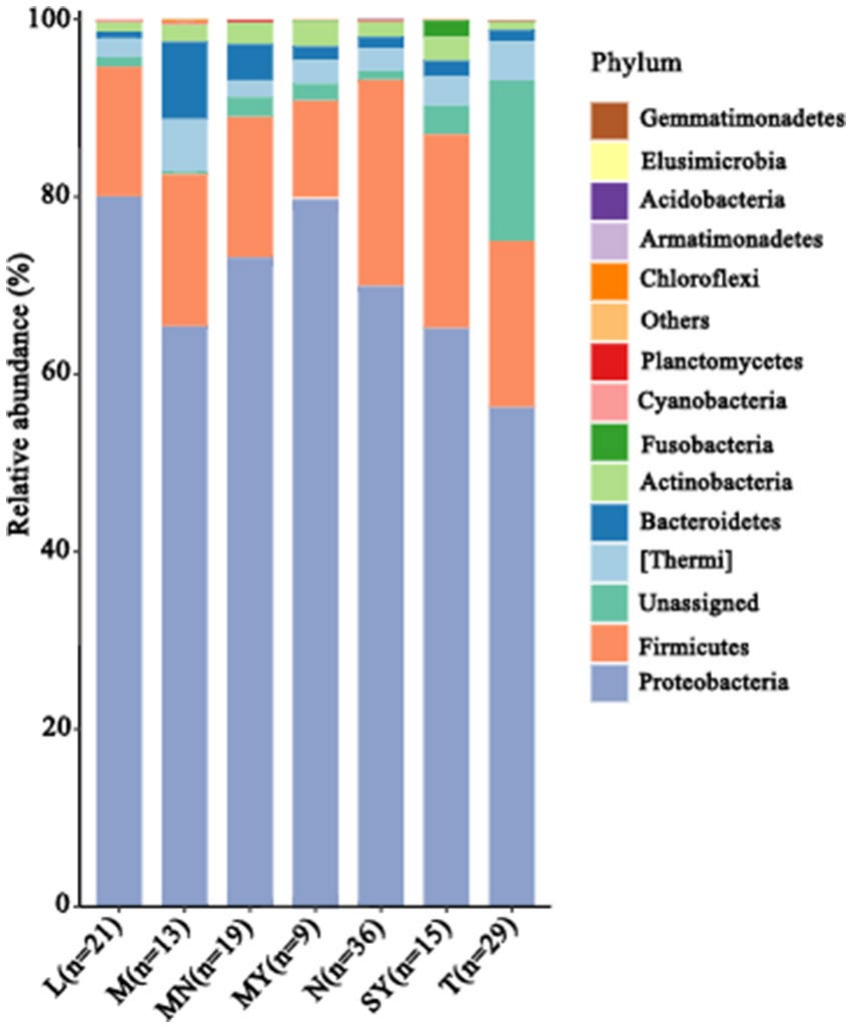
The relative abundance of the dominant species (at the genus level, relative abundance >0.5%) in 142 animal raw samples of seven different animal breeds. L, donkey milk; M, horse milk; MN, yak milk; MY, sheep milk; N, cow milk; SY, goat milk; T, camel milk.

Next, a genus with a relative abundance higher than 0.5% was defined as the predominant genus. We observed 28 predominant genera (Figure 2), and prevalent genera were diverse across the groups. The dominant bacterial genera shared by groups M and MN were *Amycolatopsis*, *Ralstonia*, *Methylobacterium*, *Bradyrhizobium*, *Propionibacterium*, *Sphingomonas*, *Phyllobacterium*, *Sediminibacterium*, and *Ochrobactrum*. Additionally, group M contained the following prevalent microbial genera: *Delftia*, *Tepidimonas*, *Hydrogenophaga*, *Anoxybacillus*, *Staphylococcus*, *Flavobacterium*, *Thermus*, and *Lactobacillus*, whereas group MN contained the following prevalent microbial genera: *Enhydrobacte*, *Streptococcus*, *Microbacterium*, and *Delftia*. The dominant bacterial genera in group T were as follows: *Acetobacter*, *Acinetobacter*, *Salinicoccus*, *Enhydrobacter*, *Leuconostoc*, *Macrococcus*, *Moraxella*, *Thermus*, and *Lactobacillus*. The dominant bacterial genera in group N were as follows: *Kocuria*, *Carnobacterium*, *Bacillus*, *Clostridium*, *Paenibacillus*, *Meiothermus*, *Psychrobacter*, and *Exiguobacterium*. The dominant bacterial genera in group L were as follows: *Erwinia*, *Lactococcus*, *Enterococcus*, *Stenotrophomonas*, *Sediminibacterium*, *Phyllobacterium*, *Lactobacillus*, *Comamonas*, and *Pseudomonas*. The dominant bacterial genera shared by groups MY and SY were as follows: *Lysobacter*, *Stenotrophomonas*, *Tepidimonas*, and *Hydrogenophaga*. Additionally, group SY contained *Moraxella*, *Brochothrix*, *Vagococcus*, *Erwinia*, *Fusobacterium*, *Porphyromonas*, *Lactococcus*, and *Enterococcus*, whereas group MY contained *Comamonas*, *Pseudomonas*, and *Corynebacterium*.

### 3.2 *α*- and *β*-diversity indices of milk microbiota

α-Diversity index indicates the microbial diversity of a given sample (Walters and Martiny, 2020). Herein, Ace, Chao, Shannon, and Simpson indices were analyzed to analyze the microbial diversity of each milk sample. Significant differences were observed among the indices of each group (Table 1). Ace, Chao, and Shannon indices of group T were significantly higher than those of the remaining groups, whereas the Simpson index of group T was significantly lower than that of the remaining groups. These results indicated that the microbial diversity of the camel milk samples was higher than that of the remaining groups.

**Table 1:** Alpha diversity indices of raw milk samples from seven distinct animal species.

Sample name	Diversity index			
	Chao1	ACE	Shannon	Simpson
L	6102.79 ± 427.05^d^	3462.09 ± 519.68^c^	2.27 ± 0.024^e^	0.31 ± 0.004^a^
M	7211.29 ± 496.19^c^	4020.16 ± 683.64^b^	2.73 ± 0.028^b^	0.25 ± 0.005^e^
MN	7943.26 ± 512.19^c^	4438.95 ± 655.38^a^	2.71 ± 0.023^b^	0.21 ± 0.003^f^
MY	8609.47 ± 536.84^b^	4677.58 ± 668.43^a^	2.40 ± 0.022^d^	0.26 ± 0.003^d^
N	5174.10 ± 350.43^e^	3282.61 ± 477.14^d^	2.53 ± 0.025^c^	0.28 ± 0.004^c^
SY	8463.74 ± 566.41^b^	4551.33 ± 736.57^a^	2.31 ± 0.022^e^	0.31 ± 0.004^b^
T	9859.15 ± 559.04^a^	5417.32 ± 660.46^a^	2.78 ± 0.021^a^	0.20 ± 0.002^g^

The abbreviations mean donkey milk (L), horse milk (M), yak milk (MN), sheep milk (MY), cow milk (N), goat milk (SY), and camel milk (T). Different lowercase letters indicate a significant difference between groups, while the same lowercase letter indicates no significant difference between groups (Tukey’s test, *p* < 0.05).

**Figure 3:**
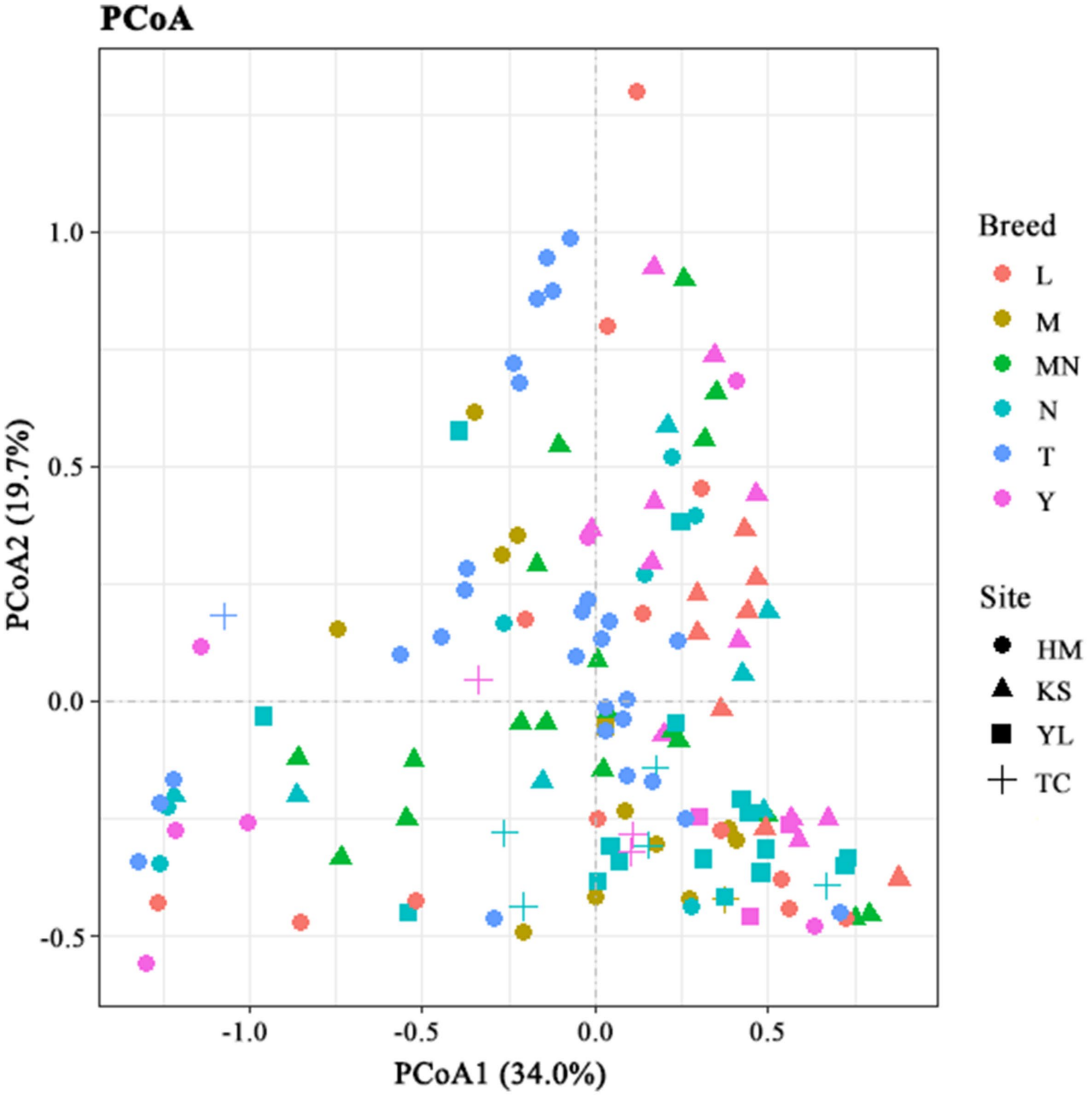
Principal coordinate analysis of bacterial communities based on genus level. L, donkey milk; M, horse milk; MN, yak milk; Y, goat or sheep milk; N, cow milk; T, camel milk; HM, Hami; TC, Tarbagatay; KS, Kashgar; YL, Ili.

The β-diversity index indicates the microbial diversity between different samples (Walters and Martiny, 2020). To compare similarities in microbial compositions, we performed PCoA using genus-level taxonomic profiles. The clustering of the milk samples according to their microbiota helped separate the samples (Figure 3). However, no clear separation was observed for milk samples from groups HM, TC, KS, and YL (different regions) and groups N, MY, SY, L, M, T, and MN (different breeds) within the PCoA plots. These results indicated that complex factors might affect microbial community structures in raw milk, and only one factor (breed or region) might not be sufficient to determine microbial community structures in raw milk. When the two factors, breed, and region, were combined some regular patterns were observed in the PCoA plots (Figure 3). Homologous animal milk samples from the same region showed a higher probability of gathering. For instance, values for the cow milk samples from Kashgar were gather together in the plots, indicating a similar microbial community structure in these samples. Similarly, values for the cow milk samples from Ili were gather together in the plots. Additionally, values for different animal milk samples from the same region were observed to be gather together. For example, values for the cow, sheep, and horse milk samples were at a shorter distance than those for the remaining samples in the plots, which suggested a similarity in these samples. Sometimes, values for the raw milk samples from the same region and breed were not gather together, which indicated that microbial communities in these samples differed from each other. This case was observed in the camel milk samples from Hami and the yak milk samples from Kashgar with large dispersion.

### 3.3 Microbial composition differences among different animal raw milk

#### 3.3.1 Microbial composition differences among donkey, horse, camel, and yak milk

**Figure 4:**
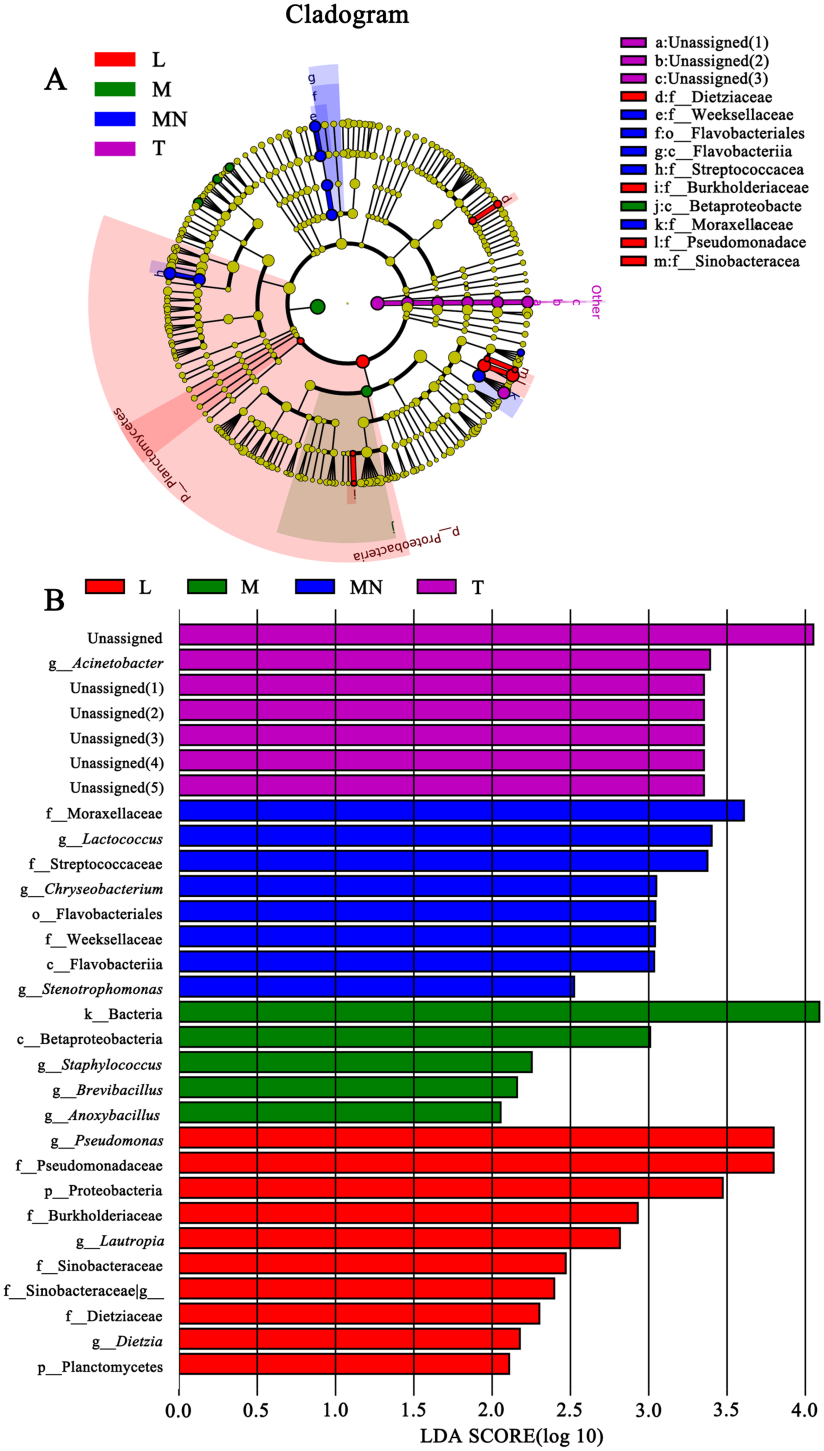
Cladogram **(A)** and LDA score **(B)** of LEfSe analysis of bacterial among seven different breed raw milk. Only the taxa with meeting a significant LDA threshold value of >2 and/or < −2 were shown. L, donkey milk; M, horse milk; MN, yak milk; T, camel milk.

**Figure 5:**
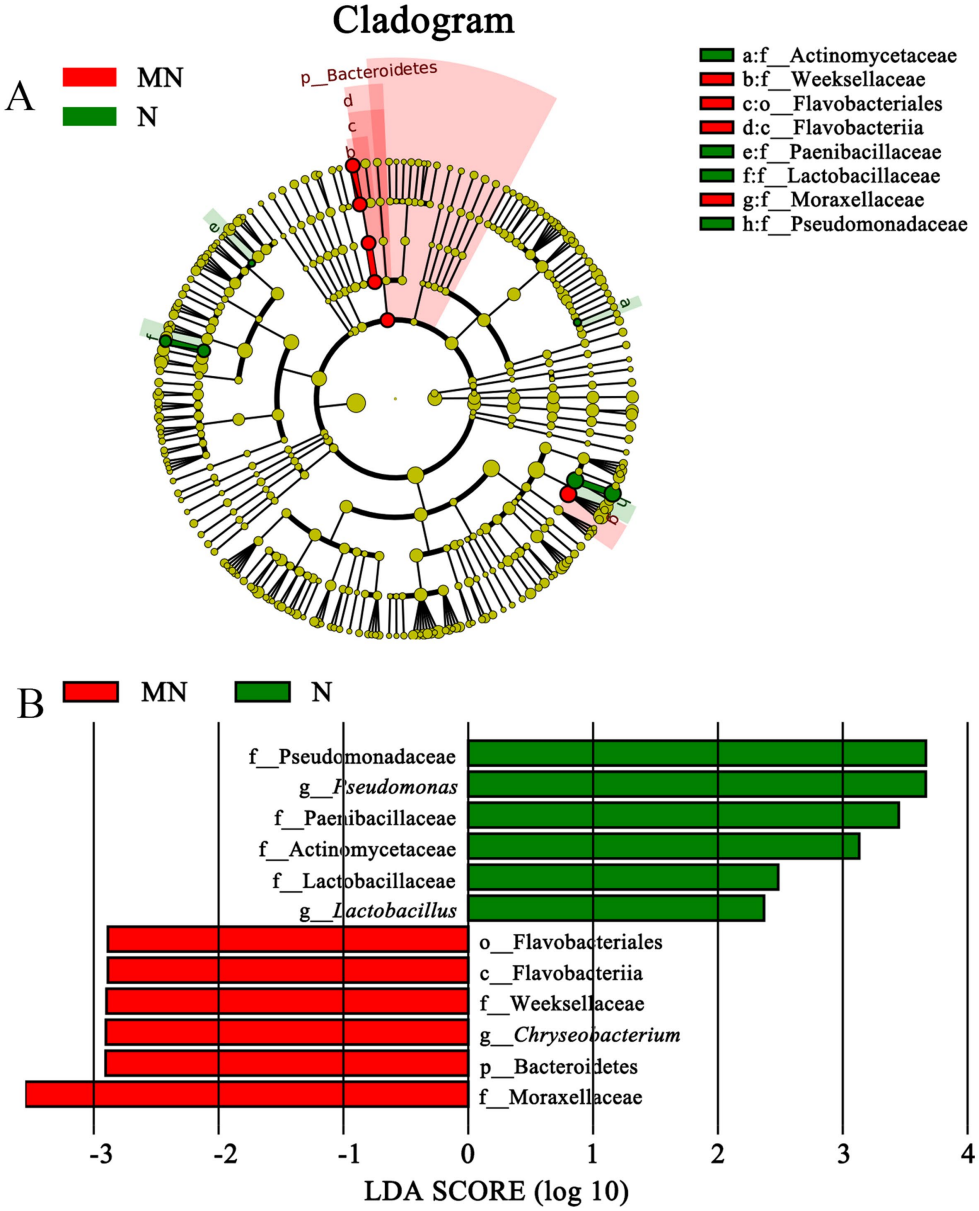
Cladogram **(A)** and LDA score **(B)** of LEfSe analysis of bacterial between yak milk and cow milk. Only the taxa with meeting a significant LDA threshold value of >2 and/or < −2 were shown. M, horse milk; MN, yak milk.

**Figure 6:**
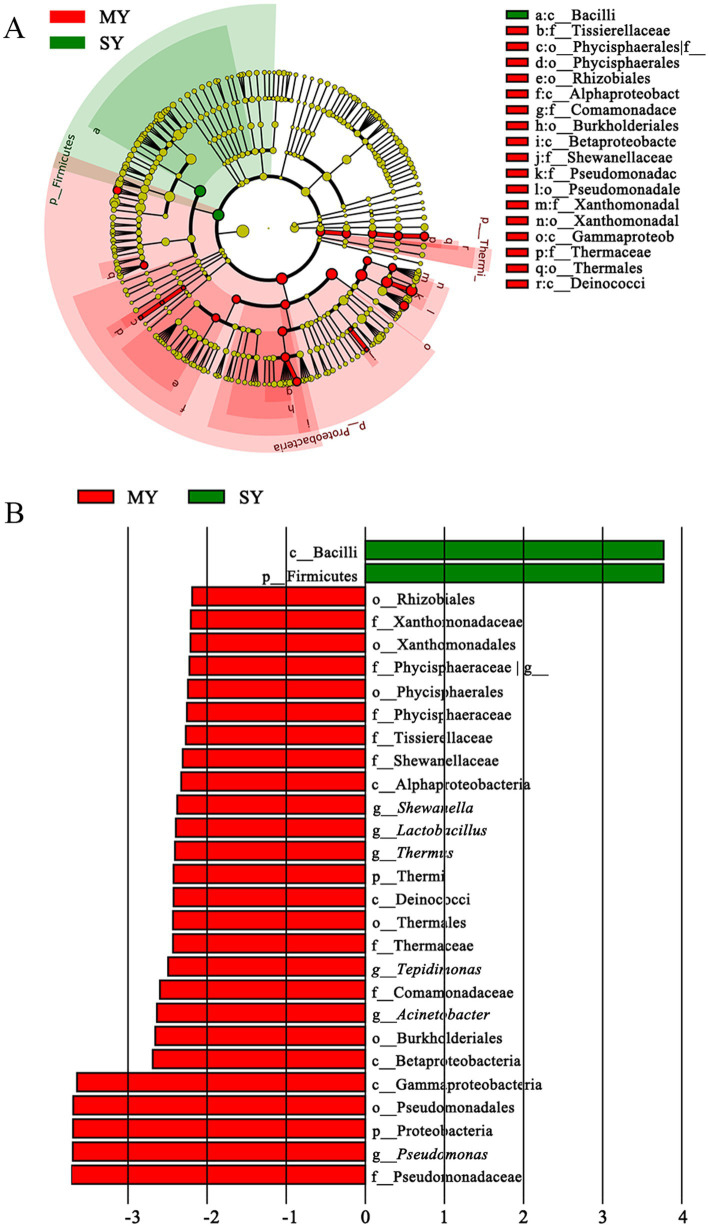
Cladogram **(A)** and LDA score **(B)** of LEfSe analysis of bacterial between sheep milk and goat milk. Only the taxa with meeting a significant LDA threshold value of >2 and/or < −2 were shown. MY, sheep milk; SY, goat milk.

To identify differences in the microbial compositions in the milk samples among donkey, horse, camel, and yak milk, we performed LEfSe in order to identify biomarkers at genus- and phylum levels with an LDA score of more than 2.0 (*p* < 0.05). In total, 44 bacterial groups were statistically significantly different, whereas 30 bacterial groups with LDA > 2.0 were selected. Four families (Dietziaceae, Burkholderiaceae, Pseudomonadace, and Sinobacteracea) were significantly enriched in group L, whereas only one class (Betaproteobacte) was significantly enriched in group M. A bacterial lineage and two families enriched in group MN were Flavobacteriia (the class, its order Flavobacteriales, and its family Weeksellaceae) and Streptococcacea and Moraxellaceae, respectively. A genus (*Acinetobacter*) and unassigned bacteria were significantly abundant in group T (Figures 4A,B). No statistically significant difference (LDA score > 2.0, *p* < 0.05) was observed in groups N and Y (sheep or goat), which was attributed to their great internal differences. Additionally, comparisons were performed between groups MN and N (Figure 5) and between groups MY and SY (Figure 6) to identify the microbial community characteristics of groups N and Y.

#### 3.3.2 Microbial composition differences among cow and yak milk

Moraxellaceae (family) and Bacteroidetes (the Phylum, its class Flavobacteriia, its order Flavobacteriales, its family Weeksellaceae, and its genus *Chryseobacterium*) were significantly more enriched in group MN than in group N, whereas four families (Actinomycetaceae, Paenibacillaceae, Lactobacillaceae, and Pseudomonadaceae) and two genus (*Lactobacillus* and *Pseudomonas*) were significantly enriched in group N (Figure 5). The comparison between groups MN and N showed that pathogenic bacteria, including *Chryseobacterium*, were more prevalent in group MN, whereas probiotic LAB, including *Lactobacillus*, were more prevalent in group N.

#### 3.3.3 Microbial composition differences among goat and sheep milk

The comparison between groups MY and SY showed that only the Firmicutes phylum (the phylum and its class Bacilli) was significantly enriched in group SY, whereas Gammaproteobacteria (Xanthomonadales, *Shewanella*, *Pseudomonas*, *Acinetobacter*), Thermi (*Thermus*), Firmicutes (*Lactobacillus*, Tissierellaceae), Planctomycetes (Phycisphaerales), Alphaproteobacteria (Rhizobiales), and Betaproteobacteria (*Tepidimonas*) were more enriched in group MY (Figure 6). This result indicated that nearly half of the predominant bacterial phyla were different between the two groups.

### 3.4 The co-occurrence of bacteria in different animal milk based on genus level

**Figure 7:**
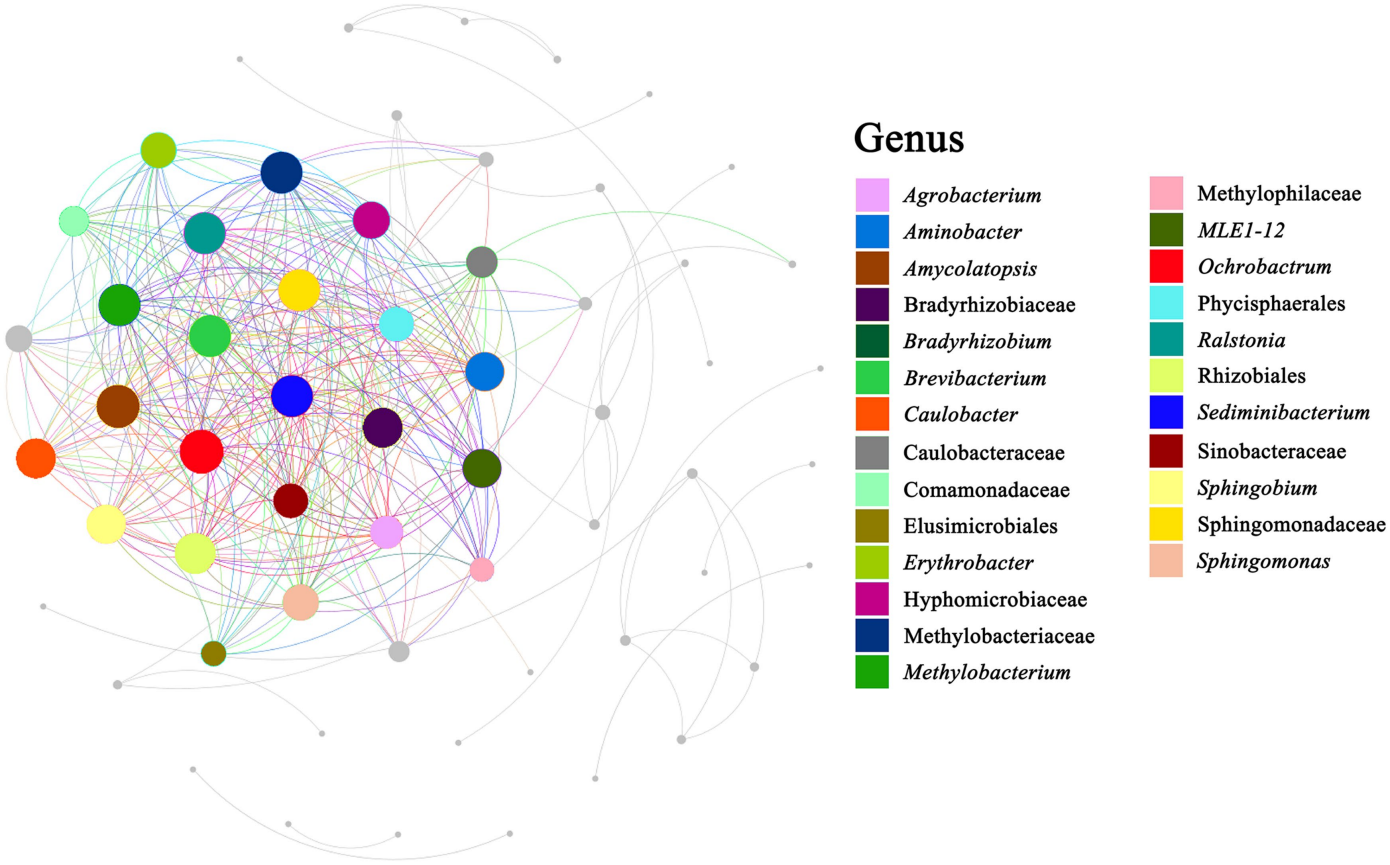
Correlation network diagram. Summary of the significant (*p* ≤ 0.05) interactions among bacterial (at genus level).

Co-occurrence network analysis was performed to determine potential relationships among bacterial genera in the milk samples. The co-occurrence network comprised 39 nodes and 68 edges, with 54 positive and 14 negative correlations (*p* < 0.05) (Figure 7). Three genera, namely *Pseudomonas*, *Lactococcus*, and *Anoxybacillus*, with more neighboring connections, were defined as the core points. Among the three core points, *Pseudomonas* showed the most neighboring connections. Two genera (*Lysobacter* and *Tepidimonas*) showed a significant positive correlation (*p* < 0.001) with *Pseudomonas*, whereas three genera (*Lactococcus*, *Enhydrobacter*, and *Stenotrophomonas*) showed a significant negative correlation (*p* < 0.001) with *Pseudomonas*. The reason behind this may be the antagonism between *Lactococcus* and *Pseudomonas* (Beeram and Silpa, 2021). *Lactococcus* showed a significant positive correlation with *Erwinia*, suggesting that these two genera were likely to share a symbiotic or syntrophic relationship. Conversely, *Lactococcus* showed a significant negative correlation with seven genera, namely *Ochrobactru*, *Tepidimonas*, *Delftia*, *Comamonas*, *Phyllobacterium*, and *Propionibacterium*, indicating a probably antagonistic relationship between *Lactococcus* and these genera. *Anoxybacillus* showed a significant positive correlation with many genera such as *Ochrobactrum*, *Thermus*, *Tepidimonas*, *Delftia*, *Sediminibacterium*, *Ralstonia*, *Agrobacterium*, *Amycolatopsis*, *Meiothermus*, and *Sphingomonas*, suggesting that these genera were likely to share a symbiotic or syntrophic relationship with *Anoxybacillus*. Additionally, the presence of *Bacillus* was negatively correlated with the presence of *Amycolatopsis*, *Sphingomonas*, *Bradyrhizobium*, and *Propionibacterium*, whereas the presence of *Enhydrobacter* was negatively correlated with the presence of *Streptococcus*, *Leuconostoc*, *Chryseobacterium*, *Deinococcus*, and *Kocuria*. Although the co-occurrence network sheds light on the complex relationships among the raw milk microbiota, empirical evidence is needed to support their natural presence.

### 3.5 Microorganisms indicator species among different breeds animal milk

To determine the existence of indicator species, INDVAL analysis, which identifies the indicator species based on OTU fidelity and relative abundance, was run by using the dataset OTU within the R environment. Only OTUs with significant (*p* < 0.05) INDVAL values that were > 0.3 were considered, as the latter value can be regarded as a good threshold for habitat specialization (Cáceres and Legendre, 2009). Among the 6,452 OTUs, indicator species analysis revealed 1755 OTUs significantly (*p* < 0.05) associated with the breed when computed across a Seven-group combination. The indicator bacteria assigned at different taxonomic levels are presented in supporting information (Supplementary Table S2).

Nine OTUs exhibited common characteristics in cattle milk (group N) and they belonged to Firmicutes (*Clostridium, Enterococcus, Weissella, Brochothrix*, and *Actinobacteria*). Among them, *Clostridium* and *Brochothrix* (spoilage microorganisms) and *Arcanobacterium* (pathogenic microorganisms) deserve more attention (Hijazin et al., 2012; Gribble and Brightwell, 2013; Deslauriers et al., 2024; Yang et al., 2024). In addition, 32 OTUs were characteristic of donkey milk (group L) and belonged to Corynebacterium (*Actinobacteria*), three genera of Firmicutes (*Clostridium*, *Streptococcus,* and *Tepidibacter*), and six genera of Proteobacteria (*Pseudomonas*, *Moraxella*, *Rhodobacter*, *Stenotrophomonas*, *Pseudomonas*, *Stenotrophomonas*). Among them, prevention against contamination by pathogenic microorganisms (such as *Corynebacterium, Streptococcus, Pseudomonas,* and *Moraxella*) during the production process is very important (Soucek et al., 1965; Potgieter and Chalkley, 1991; Ding et al., 2024; Subsomwong et al., 2024).

A total of 220 OTUs displayed a significant association with the horse milk (group M) and belonged to Acidobacteria (*Actinomyces*, *Actinomycetospora*, *Arsenicicoccus Corynebacterium*, *Propionibacterium*), Bacteroidetes (*Flavobacterium*, *Rudanella*, *Chryseobacterium*, *Bacteroides*), Firmicutes (*Allobaculum, Anoxybacillus*, *Bacillus*, *Brevibacillus*, *Geobacillus*, *Granulicatella*, *Lactobacillus*, *Lactococcus*, *Leuconostoc*, *Macrococcus*, *Paenibacillus*, *Paenibacillus*, *Planomicrobium*, *Rummeliibacillus*, *Staphylococcus*, *Streptococcus*), Planctomycetes (*Gemmata*), Proteobacteria (*Acetobacter*, *Hyphomicrobium*, *Brevundimonas*, *Methylobacterium*, *Pseudochrobactrum* belong to the class Alphaproteobacteria; *Thiobacillus* and *Hydrogenophaga* belong to the class Betaproteobacteria; *Desulfobacca* and *Anaeromyxobacter* belong to the class Deltaproteobacteria; *Acinetobacter*, *Pseudoalteromonas*, *Pseudomonas*, and *Serratia* belong to the class Gammaproteobacteria). Among them, contamination with pathogenic microorganisms (*Arsenicicoccus*, *Propionibacterium*, *Chryseobacterium*, *Granulicatella*, *Streptococcus*) should be prevented during the production process (Jeong et al., 2021; Tahir et al., 2023; Chamlagain et al., 2024; Genco et al., 2024; Yu et al., 2024).

A total of 79 OTUs affiliated to Actinobacteria (Microbacterium, Luteococcus, Brachybacterium, Microbacterium, Brachybacterium, Yonghaparkia), Bacteroidetes (Prevotella, Chryseobacterium, Bacteroides), Firmicutes (Streptococcus, Faecalibacterium, Macrococcus, Lactococcus), Betaproteobacteria (Tepidimonas, Hydrogenophaga, Ralstonia), and Gammaproteobacteria (Acinetobacter, Enhydrobacter, Mannheimia, Moraxella, Pseudomonas) were associated with the yak milk (MN). Among them, Luteococcus, Hydrogenophaga, and Acinetobacter are reported to be pathogenic, and steps should be taken to prevent their growth (Vieu et al., 1980; Okiki Pius et al., 2015; Feichtinger et al., 2023).

A total of 189 OTUs affiliated to Actinobacteria (Agrococcus, Brachybacterium, Collinsella, Corynebacterium, Dietzia, Leucobacter, Nocardioides), Bacteroidetes (Prevotella, Chryseobacterium, Porphyromonas, Adhaeribacter), Firmicutes (Aerococcus, Alkalibacterium, Ammoniphilus, Anaerococcus, Bacillus, Butyrivibrio, Dialister, Enterococcus, Gallicola, Jeotgalicoccus, Lactobacillus, Mogibacterium, Planococcus, Planomicrobium, Ruminococcus, Salinicoccus, Streptococcus), Fusobacteria (Fusobacterium), Alphaproteobacteria (Methylopila, Devosia, Brevundimonas), Betaproteobacteria (Lautropia, Hydrogenophaga, Lautropia, Azoarcus), and Gammaproteobacteria (Acinetobacter, Alcanivorax, Enhydrobacter, Halomonas, Luteimonas, Lysobacter, Pseudomonas, Pseudoxanthomonas) were characteristic of sheep milk (group MY). Among them, Collinsella, Leucobacter, Prevotella, Alkalibacterium and Lactobacillus are reported to be beneficial bacteria (Yumoto et al., 2008; Shu et al., 2013; Gomez-Arango et al., 2018; Chang et al., 2019; Jiang et al., 2024), while Corynebacterium, Chryseobacterium, Porphyromonas, Adhaeribacter, Dialister, Streptococcus, Fusobacteria, and Lautropia are reported to be harmful microorganisms (Soucek et al., 1965; Potgieter and Chalkley, 1991; Harrandah et al., 2021; Calheiros Cruz et al., 2022; Antonyuk et al., 2023; Mena-Vázquez et al., 2023; Genco et al., 2024).

A total of 39 OTUs displayed a significant association with goat milk (group SY): Actinobacteria (*Sanguibacter* and *Arthrobacter*), Bacteroidetes (*Porphyromonas*) Firmicutes (*Enterococcus*), Gammaproteobacteria (*Pseudomonas*, *Erwinia*, *Pseudomonas*), and Betaproteobacteria (*Tepidimonas*). Among them, *Erwinia* (spoilage microorganisms), *Porphyromonas,* and *Pseudomonas* (pathogenic microorganisms) deserve special attention (Kang et al., 2023; de Jongh et al., 2024; Subsomwong et al., 2024).

A total of 281 OTUs demonstrated a significant association with camel milk (group T): Actinobacteria (*Corynebacterium*, *Leucobacter*, *Nesterenkonia*, *Cryocola*, *Slackia*, *Microbacterium*), Bacteroidetes (*Bacteroides*, *Capnocytophaga*, *Chryseobacterium*, *Ornithobacterium*, *Paludibacter*, *Porphyromonas*, *Prevotella*, *Riemerella*, *Wautersiella*), Firmicutes (*Bacillus*, *Butyrivibrio*, *Catonella*, *Clostridium*, *Coprobacillus*, *Coprococcus*, *Dorea*, *Facklamia*, *Filifactor*, *Fusibacter*, *Helcococcus*, *Lactococcus*, *Paenibacillus*, *Streptococcus*, *Tissierella_Soehngenia*), Fusobacteria (*Leptotrichiaceae*, *Fusobacteriaceae*, *Leptotrichiaceae*), Alphaproteobacteria (*Caulobacterales*, *Sphingomonadales*, *Rhizobiales*, *Rickettsiales*), Betaproteobacteria (*Neisseriales*, *Burkholderiales*, *Rhodocyclales*), Epsilonproteobacteria (*Campylobacter*), Gammaproteobacteria (*Acinetobacter*, *Aggregatibacter*, *Erwinia*, *Halomonas*, *Klebsiella*, *Moraxella*, *Pasteurella*, *Pseudomonas*, *Stenotrophomonas*), and Spirochaetes (*Treponema*). Among these, *Corynebacterium*, *Chryseobacterium*, *Porphyromonas*, *Riemerella*, *Facklamia*, *Rickettsiales*, *Neisseriales*, *Burkholderiales*, *Campylobacter*, *Acinetobacter*, *Aggregatibacter*, *Erwinia*, *Klebsiella*, *Moraxella*, *Pasteurella*, and *Pseudomonas* have been reported to be harmful (Soucek et al., 1965; Lu et al., 2019; Zou et al., 2020; Wen et al., 2021; Awad et al., 2022; Pérez-Cavazos et al., 2022; Reina et al., 2022; Gloanec et al., 2023; Kang et al., 2023; Li et al., 2023; Lu et al., 2023; Mason et al., 2023; Othman et al., 2023; de Jongh et al., 2024; Genco et al., 2024). Meanwhile, these bacteria have a wide niche breadth and are considered habitat generalists.

When computed across the four-group combination: HM (Hami), TC (Tarbagatay), KS (Kashgar), and YL (Ili), the indicator species analysis revealed 146 OTUs that were significantly (*p* < 0.05) associated. The indicator bacterial assigned at different taxonomic levels are reported in supporting information (Supplementary Table S3). Only 1 indicator species, *Saccharomonospora* (Actinobacteria) was found to be associated with *Kashgar* (group KS). Group HM (*Hami*) was characterized by Firmicutesc (*Brevibacillus*), Bacteroidetesc (*Prevotella, Ornithobacterium, Riemerella*), and Proteobacteriac (*Xanthobacter*). Among them, prevention against contamination by pathogenic microorganisms (such as *Ornithobacterium* and *Riemerella*) during the production process is very important (Awad et al., 2022; Liang et al., 2024). Group TC (Tarbagatay) were characterized by Actinobacteriac (Microbacteriaceaeg, Williamsiaceaeg), Bacteroidetesc (Flectobacillus, Spirosoma, Runella, Myroides), Firmicutesc (Paenibacillus, Aerococcus, Weissella, Erysipelothrix, Alicyclobacillus, Saccharibacillus, Oribacterium), Alphaproteobacteriao (Hyphomonas, Beijerinckia), Betaproteobacteria (Methyloversatilis, Schlegelella, Hydrogenophilus, Neisseria), Gammaproteobacteria (Cardiobacterium), and Synergistetesc (TG5). Among them, Alicyclobacillus (spoilage microorganisms), Erysipelothrix, Hyphomonas, Hydrogenophilus, and Neisseria (pathogenic microorganisms) deserve special attention (Nguyen et al., 2019; Fukui et al., 2023; Kapat et al., 2023; Liu et al., 2023; Liyayi et al., 2023). Group YL (Ili) were characterized by Actinobacteria (*Yaniella*, *Trueperella*, *Serinicoccus*), Bacteroidetes (*Sporocytophaga*), Chloroflexi (Ardenscatena), Firmicutes (Lactobacillaceae, *Catenibacterium*, Tissierellaceae, *Pseudoramibacter*), Planctomycetes (*Pirellula*, *Nostocoida*, *Lautropia*, *Citrobacter*, *Nannocystis*, *Syntrophobacter*, *Sinorhizobium*), and Verrucomicrobiac (*Chthoniobacter*). Among these, *Trueperella*, Bacteroidetes, and *Citrobacter* have been reported to be harmful (Patrick, 2022; Stuby et al., 2023; Liu et al., 2024).

## 4 Discussion

Herein, we evaluated bacterial communities present in raw milk obtained from seven animals from four regions in Xinjiang, China. The results suggested that the structures of these communities were affected by multiple factors rather than a single factor. Moreover, the diversity of microbial populations present in the milk samples was affected by various complex factors, such as breeds and regions, which contributed to variations in microbial community structures (Wei et al., 2021).

### 4.1 Factors affecting microbial communities in raw milk

Microbial community structures in various animal species were initially determined based on their respective growth environments. Nevertheless, the survival of microorganisms in raw milk depends on the nutritional content of the raw milk and the competition and synergy between microorganisms present in it (Mallet et al., 2012; Quigley et al., 2013; Li et al., 2018). Eventually, microorganisms establish a state of equilibrium within microecological environment of raw milk and give rise to complex microbial communities (Li et al., 2018), in which, deterministic and stochastic processes are distinguished (Keady et al., 2023). However, the variety and composition of raw milk samples and interactions among microorganisms present in the samples ultimately determine whether microorganisms from the outside environment can stably exist in the raw milk environment (Wei et al., 2021; Celano et al., 2022).

The present results indicated that animal species significantly affected the community structures of microbes present in the raw milk samples, and the reason was differences in the living environment developed in the raw milk samples obtained from different animal species for the survival of these microorganisms, including differences in the composition and physical and chemical indices of the milk samples (Moossavi et al., 2019; Albonico et al., 2020; Massouras et al., 2020). The compositions of different raw milk samples were correlated with the corresponding characteristics of microbial community structures. Moreover, the microbial community structure of the raw milk samples obtained from different animal breeds was related to some components in the samples.

#### 4.1.1 The effects of ingredients in raw milk on the microbial community

Donkey and mare milk are relatively high in lactose, promoting the proliferation of probiotics including LAB. We found relatively high levels of LAB such as *Lactobacillus* in donkey and mare milk. Donkey milk is considered the best growth medium for some useful strains of lactobacillus, and lysozyme is regarded as an indirect “bifidogenic factor” (Modler, 1994).

The concentration of lysozyme in donkey milk was significantly higher than in other animal milk. Furthermore, the proportion of lysozyme in donkey whey protein was 21.03%, which is much higher than that in horse and cow milk (Salimei et al., 2004). The high concentration of lysozyme in donkey milk contributes to its strong antibacterial activity against *Listeria monocytogenes* and *Staphylococcus aureus* due to the abundant presence of antibacterial components, particularly whey proteins such as lysozyme and lactoferrin (Derdak et al., 2020). Furthermore, donkey milk is safer and less prone to contamination by food-borne pathogenic bacteria, thus having a longer shelf life (Derdak et al., 2020).

The antibacterial property of lysozyme contributes to the simplicity of the microbial structure in donkey milk, resulting in the lowest alpha diversity (Table 1). Lysozyme can effectively inhibit various types of bacteria, including Gram-positive bacteria (*Staphylococcus aureus*, *Bacillus subtilis*, and *Streptococcus mutans*), Gram-negative bacteria (*Escherichia coli* and *Pseudomonas aeruginosa*), and (*Candida albicans*). Therefore, these pathogenic microorganisms are suppressed in donkey milk. Furthermore, donkey milk contains relatively high levels of lactose and lysozyme, promoting the dominance of LAB, including *Enterococcus*, *Sediminibacterium*, and *Lactobacillus* (Figure 2).

#### 4.1.2 Effect of microbial interactions on the final microbial community structure of raw milk

The components of raw milk greatly affect the microbial community, which is also influenced by the network relationship between microorganisms and the synergy and antagonism among them.

Various microorganisms can enter raw milk, adapt to its environment, and interact with each other. Furthermore, some microorganisms exhibit a synergistic effect. Network correlation analysis showed a significant positive correlation between *Pseudomonas*, *Tepidimonas*, and *Lysobacter*. We also found the presence of these three bacterial genera in goat and sheep milk. The dominant intestinal flora found in cheese, dairy products, and human skin are *Thermus*, *Tepidimonas*, *Delftia*, *Comamonas*, *Phyllobacterium,* and *Propionibacterium*. Notably, a significant negative correlation is present between these flora because *Lactococcus* can inhibit the growth of these spoilage and pathogenic bacteria. We found that raw milk contains abundant lactobacillus (*Leuconostoc*, *Lactobacillus*, and *Lactococcus*), such as camel milk, goat milk, and donkey milk, has relatively lower levels of spoilage bacteria and pathogenic bacteria.

The relatively low species and abundance of putrefactive bacteria in camel milk contribute to its richness in LAB, such as *Leuconostoc Lactobacillus*, and *Acetobacter*.

Donkey milk contains more lysozyme and LAB (*Lactococcus* and *Lactobacillus*) and also contains relatively few spoilage and pathogenic bacteria.

We found a significant positive correlation between many spoilage and pathogenic bacteria. For instance, *Anoxybacillus* exhibited a significant positive correlation with *Ochrobactrum*, *Delftia*, *Ralstonia*, and *Sphingomonas.* These spoilage and pathogenic bacteria are found in horse and yak milk.

To summarize, complementary metabolism and synergistic or antagonistic effects occur among the microorganisms present in raw milk. The removal and retention of microorganisms in raw milk are determined by their interaction and balance, which ultimately form the microbial community structure in raw milk.

### 4.2 Probiotic resources and potentially harmful bacteria analysis in raw milk

#### 4.2.1 Beneficial bacterial resources

The lactose content in donkey and horse milk is relatively high, resulting in the presence of abundant *Lactobacillus*. Furthermore, donkey, camel, and goat milk contain abundant *Lactococcus*, whereas camel and yak milk have relatively rich *Leuconostoc*. These LAB can be used as starter cultures or probiotic strains.

#### 4.2.2 Potential risk

The presence of potentially pathogenic bacteria in raw milk is a risk for traditionally fermented dairy products. Therefore, precautions must be taken to prevent and control spoilage and pathogenic bacteria when using different dairy products. Our data analysis showed that horse and yak milk have a higher relative abundance of potential spoilage and pathogenic bacteria. Raw horse and yak milk contain the following bacteria: *Bradyrhizobium*, *Chryseobacterium*, *Propionibacterium*, *Sphingomonas*, and *Ochrobactrum*. Among them, *Bradyrhizobium* can lead to the deterioration of dairy products and the production of harmful substances such as biogenic amines and acid substances, which can cause food poisoning and allergic reactions. *Chryseobacterium* species found in various environments can degrade hemoglobin and produce virulence enzymes, making them potential human pathogens (Mwanza et al., 2022). Additionally, *Chryseobacterium sp.* is also responsible for the premature spoilage of milk (Alothman et al., 2017). *Propionibacterium* can cause spoilage of dairy products and contribute to skin acne, causing redness and swelling. For instance, *Propionibacterium acnes* is associated with the inflammatory process of acne lesions (Brook and Frazier, 1991). The genus *Sphingomonas* contains several pathogenic organisms, such as *Sphingomonas paucimobilis*, which are associated with meningitis, peritonitis, wound infection, and other human infections (Koskinen et al., 2000). *Ochrobactrum spp.* are generally considered to have low pathogenicity, however, they are increasingly being identified as the cause of infections in individuals with a healthy immune system (Ryan and Pembroke, 2020), with *Ochrobactrum anthropi* recognized as an opportunistic pathogen in breast milk (Asaf et al., 2020).

Horse milk was found to contain *Staphylococcus* and *Flavobacterium*. *Staphylococci*, commonly encountered pathogens, are often present in food (Kim et al., 2021), with *Staphylococcus epidermidis* being associated with various infections (Morot-Bizot et al., 2004). *Staphylococcus aureus*, another member of the *Staphylococcus* genus, is a significant foodborne pathogen capable of producing staphylococcal enterotoxins, which can adversely affect human health (Kim et al., 2021). *Staphylococcus spp.* has also been reported as a primary pathogen in horse mastitis (Colavita et al., 2016). *Flavobacterium*, detected in horse milk, is a lipolytic bacterium that produces lipolytic enzymes, contributing to the rancidity of dairy products. Some species of *Flavobacterium*, such as *Flavobacterium meningosepticum*, have the potential to cause infections such as meningitis or endocarditis, making them human pathogens (Colavita et al., 2016; Soler et al., 2023). Certain species of *Flavobacterium* are considered pathogenic or opportunistic pathogens, causing diseases in various organisms, including plants, fish, and humans (Zamora et al., 2012).

Yak milk is found to contain *Streptococcus*, which can contribute to the decomposition of protein and fat in dairy products, leading to natural spoilage. Additionally, *Streptococcus* can cause severe infectious diseases with high morbidity and mortality rates (Nguyen et al., 2015).

Camel milk and goat milk are found to contain more *Moraxella* species. *Moraxella spp.* is associated with meat spoilage. Additionally, camel milk showed a relatively higher abundance of *Acinetobacter* species. *Acinetobacter* is an opportunistic pathogen known to cause infections in immunocompromised individuals. It is considered an important opportunistic pathogen responsible for nosocomial infections. *Acinetobacter* species can colonize the digestive tract through the consumption of contaminated food. For instance, *Acinetobacter lwoffii* is believed to have the potential to induce gastritis (Carvalheira et al., 2021). Goat milk also contains a high abundance of *Brochothrix*, and *Brochothrix thermosphacta* is the main spoilage flora associated with crucian carp meat.

The problematic genera detected in the milk samples include *Kocuria*, *Carnobacterium*, and some of the *Sphingomonas Kocuria* is the main spoilage bacterium in steamed cakes. *Carnobacterium* can produce exotoxin or botulinum toxin in an anaerobic environment, which can have paralyzing effects in humans.

The genus *Pseudomonas* appears to be relatively abundant in sheep and donkey milk. Members of this genus are frequently implicated in the degradation and spoilage of a wide range of plant or animal foods (Caldera et al., 2016). Within the *Pseudomonas* genus, at least three species are known to be pathogenic to animals or humans. *Pseudomonas aeruginosa* is considered a conditional pathogen, which is typically associated with infections such as wound infections from severe burns, middle ear infections, urinary tract infections, and even sepsis.

The results of indicator bacteria analysis indicate the need to be cautious regarding the presence of potentially harmful bacteria in raw milk from various sources. It is crucial to implement appropriate measures during the production process to prevent the growth of spoilage and pathogenic bacteria.

## 5 Conclusion

To conclude, our study revealed the bacterial communities present in the raw milk of seven animals across four regions in Xinjiang, China. Camel milk exhibited the highest bacterial diversity, accompanied by a notable presence of unidentified bacteria. The structure of bacterial communities in raw milk samples is influenced by the components of raw milk and microbial interactions. Horse and yak milk showed a higher prevalence of spoilage microorganisms and pathogenic bacteria, whereas goat, donkey, and camel milk exhibited a higher abundance of probiotic LAB. Furthermore, indicator species analysis showed that raw milk from each breed and every region contained specific pathogenic microorganisms that should be given attention and their presence should be prevented. The study contributed to the further development and use of different animal raw milk while providing help to prevent and control the presence of pathogenic microorganisms in the production process. Nevertheless, further investigations are warranted to determine the underlying reason for the common habitat selection of bacteria and their communications.

## Data Availability

The datasets presented in this study can be found in online repositories. The names of the repository/repositories and accession number(s) can be found below: NCBI -PRJNA1170235, SAMN44099913 - SAMN44100054.
