# Supplementary figures and images for: Insights into the microbiota of raw milk from seven breeds animals distributing in Xinjiang China
Source: Front Microbiol. 2024 Oct 23;15:1382286. doi: 10.3389/fmicb.2024.1382286 (PMC11537933; doi:10.3389/fmicb.2024.1382286)

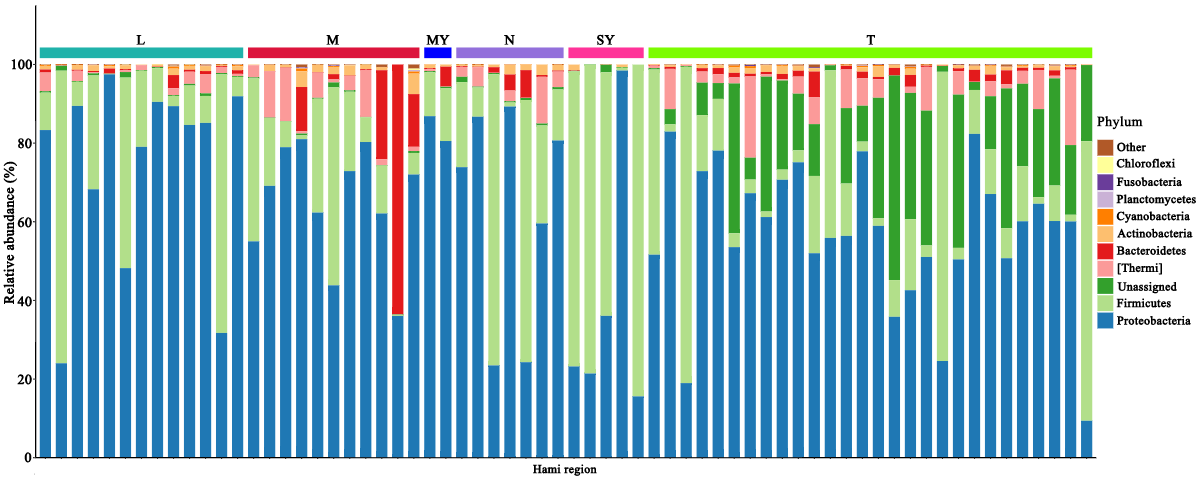

Supplement: Supplementary file 5 [file Image_1.TIF]

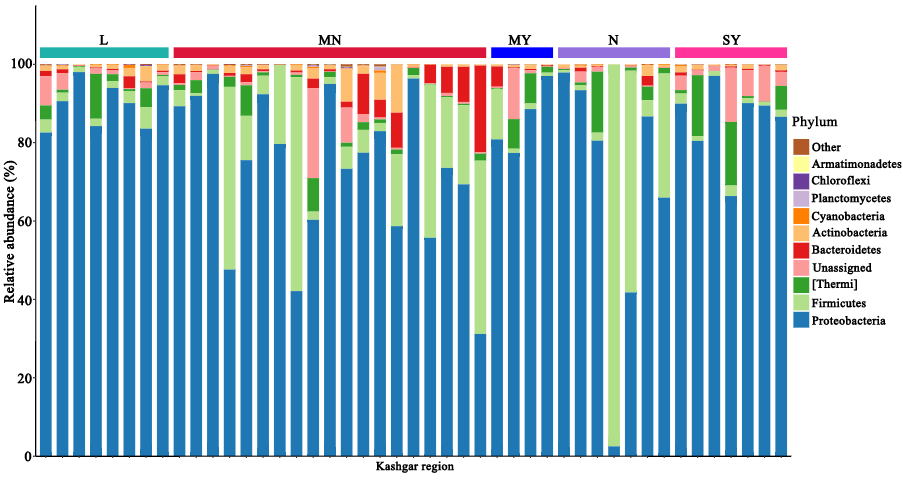

Supplement: Supplementary file 6 [file Image_2.TIF]

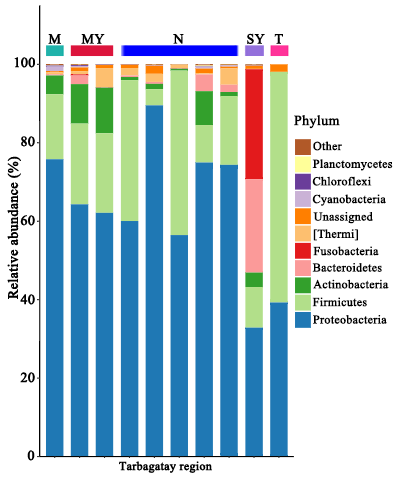

Supplement: Supplementary file 7 [file Image_3.TIF]

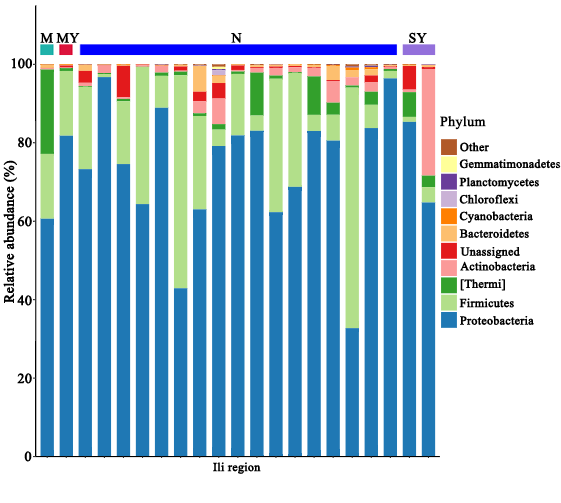

Supplement: Supplementary file 8 [file Image_4.TIF]
